# Pulmonary Microvascular Endothelial Antigen Presentation Activates resident CD8^+^ T Cells to Restrain Influenza Lung Injury

**DOI:** 10.21203/rs.3.rs-7796497/v1

**Published:** 2026-01-08

**Authors:** Yuanyun Ao, Kamal Bagale, Sophia Hu, Ahmed Mostafa, Chengjin Ye, Kienan Salvadore, Gregory Gibson, Ricardo Pineda, Jiayue Lu, Rachel Covitz, Dejuanna Chan, Ryan Langlois, James C Zimring, Paul Duprex, Claudette M St Croix, John F. Alcorn, Jalees Rehman, Melanie Koenigshoff, Luis Martinez-Sobrido, Jianhua Xing, Lianghui Zhang

**Affiliations:** 1 Division of Pulmonary, Allergy, Critical Care, and Sleep Medicine, University of Pittsburgh, Pittsburgh, PA, USA.; 2 Center for Vaccine Research, University of Pittsburgh, Pittsburgh, PA, USA.; 3 Department of Computational & Systems Biology, University of Pittsburgh, Pittsburgh, PA, USA; 4 Host-pathogen interactions and Disease Intervention and Prevention programs, Texas Biomedical Research Institute, San Antonio, TX, USA.; 5 Center for Biologic Imaging, University of Pittsburgh, Pittsburgh, PA, USA.; 6 Dept of Microbiology & Immunology, University of Minnesota, Minneapolis, MN, USA.; 7 Department of Pathology, University of Virginia, Charlottesville, VA, USA.; 8 Department of Pediatrics, UPMC Children’s Hospital of Pittsburgh, University of Pittsburgh, Pittsburgh, PA, USA.; 9 Department of Biochemistry and Molecular Genetics, University of Illinois, College of Medicine, Chicago, IL, USA.; 10 Vascular Medicine Institute, University of Pittsburgh, Pittsburgh, PA, USA.

## Abstract

The remaining unacceptably high mortality of influenza-induced acute respiratory distress syndrome underscores the urgent need to identify key cellular drivers of host responses. Endothelial cells (ECs) are increasingly recognized for their immunomodulatory roles, but whether they function as antigen-presenting cells (APCs) following respiratory viral infection remains unknown. Here, we show that influenza A virus H1N1 restrictively infects pulmonary microvascular ECs (PMVECs) during late-stage acute lung injury, triggering robust MHC class I (MHC-I) upregulation in vitro, in vivo, and in ex vivo human precision-cut lung slices. Infected PMVECs present H1N1 antigens via MHC-I and co-stimulatory CD40 to lung-resident CD8^+^ T cells, driving their proliferation and effector function (Granzyme B, IFNγ) to promote viral clearance and resolve inflammation. This process is IFNγ-dependent and STAT1-regulated, forming a positive feedback loop that enhances PMVEC antigen presentation and CD8^+^ T cells activation. By contrast, the emerging H5N1 (A/Texas/37/2024) infect pulmonary ECs earlier and more broadly but elicits weaker pulmonary EC-driven CD8+ T cell responses, potentially contributing to its higher pathogenicity. These findings reveal PMVECs as active APCs in antiviral defense and highlight new avenues for immunotherapeutic intervention.

## INTRODUCTION

Respiratory viral infections remain a leading cause of global mortality and morbidity^1,2^, primarily due to virus-induced acute lung injury and its more severe manifestation in acute respiratory distress syndrome (ARDS). Recurrent influenza epidemics and pandemics further exacerbate this burden, posing a persistent threat to global public health^3^. There are no targeted pharmacological interventions available to reduce the high mortality of respiratory virus-induced ARDS (25–40%)^4,5^, highlighting the urgent need to identify key cellular drivers of host responses and to develop novel antiviral strategies. An emerging highly pathogenic avian influenza virus (HPAIV) H5N1 crossed the species barrier to infect humans in the United States in 2024, with over 70 reported human cases and one death so far, raising concerns about its epidemic or pandemic potential^6–9^. Accordingly, understanding the mechanisms underlying the high pathogenicity of severe influenza viruses is critical for developing effective antiviral strategies.

Endothelial cells (ECs) canonically regulate immunity by recruiting and guiding the transmigration of circulating immune cells into tissues^10^. Although dendritic cells (DCs) are considered the principal antigen-presenting cells (APCs) during viral infections^11,12^, growing evidence suggests that ECs can also present antigens in diverse contexts^13^, including cancer^14^, transplant rejection^15^, liver fibrosis^16,17^, malaria^18^, and diabetes^19^. Consistent with this notion, we and others have shown that pulmonary vascular ECs (PVECs) are particularly enriched for genes involved in antigen presentation and T-cell regulation^13,20–22^. However, it remains unknown whether PVECs actively present viral antigens and shape T cell response during respiratory virus infections, such as severe influenza A virus (IAV).

Here, we demonstrate that H1N1-infected pulmonary microvascular ECs (PMVECs) during later stage of infection function as antigen-presenting cells that activate resident CD8^+^ T cells via MHC-1 and co-stimulatory CD40, promoting viral clearance through IFNγ-STAT1-dependent positive feedback loop. Furthermore, we demonstrate that the emerging HPIAV H5N1 strain (A/Texas/37/2024) exhibits broader lung endothelial tropism at earlier stages of infection and elicits weaker resident CD8^+^ T cell responses than H1N1, suggesting a key role for endothelial antigen presentation in its systemic dissemination and high pathogenicity. Our results suggest that initial CD8^+^ T cell activation may be driven by DCs, whereas sustained and prolonged T cell responses to clear viruses rely on PMVEC-mediated antigen presentation. Our findings reveal a previously unrecognized EC–T cell axis in antiviral immunity and offer a promising approach to controlling severe influenza infections, such as the emerging H5N1 “bird flu” virus.

## RESULTS

### H1N1 selectively infects PMVECs and upregulates MHC-I expression

**Figure 1.**
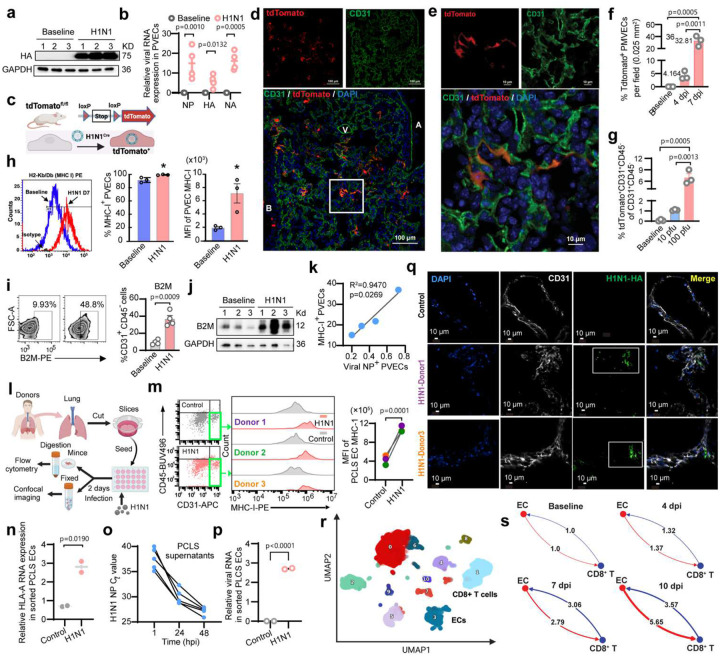
H1N1 infection of ECs restricts to PMVECs and induces MHC-I responses in mice and human PCLSs. (**a-b**, **h-k**, **r-s**) The C57BL/6 mice were intranasally inoculated with 100 PFU H1N1 (A/PR/8/1934). Lungs were harvested at indicated time points with uninfected mice as baseline. (**a-b**) Viral HA protein (Western blot) and HA, NA, NP RNA (RT-qPCR) were detected in PVECs isolated at 7 dpi. (**c-g**) tdTomato^fl/fl^ mice were inoculated with 100 PFU rH1N1^Cre^ to label virus-infected cells. (**c**) Scheme. (**d**) Representative confocal images at 7 dpi with (**e**) magnified regions highlighting tdTomato^+^ PMVECs. (**f**) Quantification of tdTomato^+^ PMVECs in infected imaging areas at 4 and 7 dpi, showed as the mean percentage of PMVECs co-localizing with tdTomato among total ECs from three randomly representative images. (**g**) Flow cytometry of tdTomato^+^ CD31^+^ CD45^−^ lung cells at 4 dpi with 10 or 100 PFU rH1N1^Cre^. (**h**) Flow plots of MHC-I expression with %MHC-I^+^ ECs and mean fluorescence intensity (MFI) at 7 dpi. (**i-j**) B2M expression in PVECs assessed by flow and Western blot at 7 dpi. (**k**) Correlation analysis between viral NP^+^ and MHC I^+^ in PVECs. (**l-q**) Human PCLSs were infected with 1×10^5^ PFU H1N1 for 48 hours. (**l**) PCLS infection scheme. (**m**) Flow plots and quantification (MFI) of HLA-ABC on PVECs from three donors (different colors). (**n**) RT-qPCR of HLA-A RNA in sorted PVECs (one donor, two replicates). Viral NP RNA was detected in PCLS supernatants (lower Ct value=higher viral load) (**o**) and sorted PVECs (**p**) (one donor, two replicates) by RT-qPCR. (**q**) Representative confocal images of H1N1-infected PMVECs. (**r-s**) single-cell RNA-seq of lung cells at baseline, 4, 7 and 10 dpi. (**r**) UMAP of lung cells with ECs and CD8^+^ T cells indicated. (**s**) CellChat analysis of EC–CD8^+^ T cell interaction strength: red – MHC-I signaling from ECs, blue - IFNγ signaling from CD8^+^ T cells; numbers and line size indicate relative strength. Data are presented as mean ± s.e.m. Statistical analysis by unpaired (b, h-i, n, p) or paired two-tailed t-tests (m) and one-way ANOVA (f-g).

H1N1 primarily infects respiratory epithelial cells, but whether it targets PVECs remains unclear. To address this, H1N1 (A/PR/8/1934, PR8) was applied to induce acute lung injury and endothelial inflammation in C57BL/6 mice at 7 days post -infection (dpi) (**Extended Data Fig. 1a-e**). H1N1 infection of PVECs was confirmed by Western Blots and RT-qPCR (Fig. 1a–b). To trace infected PVECs, we used Rosa26-tdTomato reporter mice infected with recombinant H1N1 expressing Cre recombinase (rH1N1^Cre^)^23,24^ with body weight loss comparable to PR8 (Fig. 1c, **Extended Data Fig. 1f**). Confocal imaging showed that H1N1 infection of vasculature was restricted to pulmonary microvascular ECs (PMVECs), with arteriole or venule ECs remaining intact (Fig. 1d–e, **Extended Data Fig. 1g-k**). In infected lung areas, about 32% PMVECs were infected at 7 dpi, which was significantly higher than the minimal 4% infection rate at 4 dpi (Fig. 1f), indicating preferential late-phase infection, consistent with peak lung vascular injury around 7 dpi^25,26^. Flow cytometry confirmed PMVEC infection, although the proportion of infected cells decreased from 4 to 7 dpi (**Extended Data Fig. 1l**), likely due to increased fragility or loss of virus-infected PMVECs during lung digestion. H1N1-infected PMVECs were significantly higher with 100 PFU infection than 10 PFU at 7 dpi (Fig. 1g), indicating a dose-dependent increase in infection. Next, we found that H1N1 infection significantly induced the expression of MHC-I and β_2_-microglobulin (B2M) in PVECs (Fig. 1h–j), with MHC-I levels correlating with viral presence (Fig. 1k), suggesting direct viral induction of the antigen-presentation machinery.

To further validate H1N1 infection in human PMVECs, human precision-cut lung slices (PCLSs) from three donors were infected with H1N1 (Fig. 1l–q). Flow cytometry showed a significant upregulation of MHC-I expression on PVECs across three donors (Fig. 1m), consistent with increased HLA-A RNA in sorted PVECs (Fig. 1n). Viral RNA increased in PCLS supernatants over time and in sorted PVECs (Fig. 1o–p), indicating successful PCLS and PVEC infection ex vivo. The increased MHC-1 and ICAM1expression on PVECs indicates EC activation after infection (Fig. 1m, **Extended Data Fig. 1m**). Confocal imaging further confirmed co-localization of viral HA with ECs in microvascular regions of infected PCLSs and co-localization of H1N-infected PMVECs and proliferated CD8^+^ T cells (Fig. 1q, **Extended Data Fig. 1n**). Together, our findings demonstrate that H1N1 infects human and mouse PMVECs and induces their MHC-I expression. To explore the potential interaction between PMVECs and CD8^+^ T cells, we performed single-cell RNA sequencing (sc-RNAseq) of C57BL/6 mice lung cells at baseline, 4, 7, and 10 dpi. CellChat analysis^27^ revealed progressive enrichment of MHC-I signaling from ECs to CD8^+^ T cells and IFN II signaling in the reverse direction (Fig. 1r–s, **Extended Data Fig. 2a-h**), indicating PMVECs present viral antigens to activate CD8^+^ T cells through MHC-I. To confirm this, we generated inducible endothelial-specific B2M knockout or restoration transgenic mice to investigate the role of endothelial MHC-I in the activation of CD8^+^ T cells during acute H1N1 infection.

### Endothelial MHC-I drives antiviral CD8^+^ T cell response

**Figure 2.**
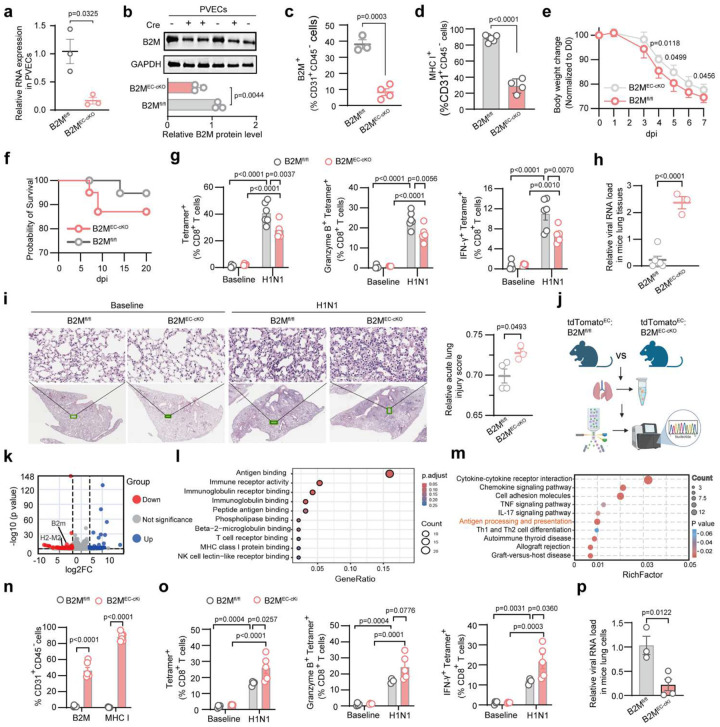
PMVEC MHC-I-mediated antigen presentation drives resident CD8^+^ T cells activation during H1N1 infection. (**a-e**) Tamoxifen-injected B2M^fl/fl^:Cdh5-Cre^ERT2^ mice were inoculated with 100 PFU H1N1. Cre^+^ mice as B2M^EC-cKO^ and Cre^−^ as B2M^fl/fl^ (controls). Lungs were harvested at baseline and 7 dpi. MHC-I (B2M, H2-Kb) express in PVECs was analyzed by RT-qPCR (**a**), Western blot (**b**) and flow cytometry (**c-d**). GAPDH as control. (**e**) Body weight and (**f**) survival were monitored. (**g**) Flow cytometry of lung-resident virus-specific CD8^+^ T cells: tetramer^+^, Granzyme B^+^ tetramer^+^, and IFNγ^+^ tetramer^+^ subsets. (**h**) RT-qPCR of H1N1 NP RNA in lung tissues, normalized to GAPDH. (**i**) Representative lung H&E staining and quantification of acute lung injury scores (ATS 2022 criteria). (**j-m**) H1N1-infected tamoxifen-treated tdTomato^fl/+^:B2m^fl/fl^:Cdh5-Cre^ERT2^ (Cre^+^) and tdTomato^fl/+^:Cdh5-Cre^ERT2^ (Cre^+^) transgenic mice. tdTomato^+^ CD31^+^ CD45^−^ were sorted by flow cytometry for bulk RNA-seq (**j**). (**k**) Differential expressed genes. (**l**) GO and (**m**) KEGG pathway enrichment analysis of downregulated genes. (**n-p**) Tamoxifen-treated endothelial-specific B2M restoration mice (B2M^inv/inv^:Cdh5-Cre^ERT2^) were inoculated with 100 PFU H1N1. Cre^+^ mice as B2M^EC-cKI^ and Cre^-^ as B2M^inv/inv^. Lungs were harvested at baseline and 7 dpi. Flow cytometry of (**n**) surface MHC-I expression (B2M and H2-Kb) of PVECs and (**o**) lung-resident virus-specific CD8^+^ T cells: tetramer^+^, Granzyme B^+^tetramer^+^, IFNγ^+^tetramer^+^ subsets. (**p**) RT-qPCR of H1N1 NP RNA in lungs, normalized to GAPDH. Data are presented as mean ± s.e.m. Statistical analysis by unpaired two-tailed t-tests (a-d, h-I, n, p) and two-way ANOVA (g, o).

B2M, a component of MHC-I complexes, is required for proper MHC-I folding and surface expression (**Extended Data Fig. 3a**). Its deletion abolishes MHC-I complex and impairs antigen presentation^28^. We generated the inducible EC-specific B2M knockout (B2m^fl/fl^:Cdh5-Cre^ERT2^) mice (**Extended Data Fig. 3b**). After tamoxifen injection, B2m^EC-cKO^ (Cre^+^) and B2m^fl/fl^ (Cre^−^) mice were inoculated with H1N1 and lungs were harvested at baseline and 7 dpi (**Extended Data Fig. 3c**). Efficient B2M deletion in PVECs was confirmed by RT-qPCR, Western blot, and flow cytometry (Fig. 2a–d), while MHC-II, ICAM1 and VCAM1 expression on PVECs were unaffected (**Extended Data Fig. 3d-e**). Following infection, B2M^EC-cKO^ mice exhibited greater body weight loss and higher mortality (Fig. 2e–f) than B2M^fl/fl^ mice. To assess the effect of EC-specific B2M deletion on lung-resident CD8^+^ T cell responses at 7 dpi, we performed flow analysis of lung resident T cell populations^29^, by gating CD45^−^ cells, labeled by retro-orbital pre-injection of anti-CD45 BV-785 to exclude intravascular immune cells in lungs^30^ (**Extended Data Fig. 3f**). No significant differences were found in total lung-resident CD8^+^ T cells and activated Granzyme B^+^ CD8^+^ T cells between B2M^EC-cKO^ and B2M^fl/fl^ mice, but IFNγ^+^ CD8^+^ T cells was significantly reduced, alongside decreased bronchoalveolar lavage fluid (BALF) CD8^+^ T cells infiltration (**Extended Data Fig. 3g-h**). Notably, virus-specific tetramer^+^ CD8^+^ T cells in lungs, including IFNγ^+^ and Granzyme B^+^ subsets, were lower in B2M^EC-cKO^ mice, correlating with higher viral loads and exacerbated lung injury (Fig. 2g–i). We further generated tdTomato^fl/+^:B2M^fl/fl^:Cdh5-Cre^ERT2^ transgenic mice to lineage trace EC with B2M deletion. The tdTomato^fl/+^:Cdh5-Cre^ERT2^ mice served as controls. The tdTomato^+^CD31^+^/CD45^−^ cells were sorted from both groups for Bulk RNA sequencing, showing that B2M deletion led to decreased expression of genes involved in antigen binding and MHC-I signaling pathways (Fig. 2j–m).

To further confirm that PMVEC MHC-I drives antiviral pulmonary resident CD8^+^ T cells, we generated conditional endothelial-specific B2M restoration (B2M^inv/inv^:Cdh5-Cre^ERT2^) mice (**Extended Data Fig. 4a**). Tamoxifen-induced recombination restored B2M to its natural orientation, re-establishing endothelial MHC-I^31^ (**Extended Data Fig. 4b**). The mice with and without Cre^ERT2^ are designated as B2M^EC-cKI^ and B2M^inv/inv^ respectively. Tamoxifen injection restored B2M, and MHC-I surface expression on PVECs without affecting MHC-II, ICAM1 or VCAM1 expressions (Fig. 2n, **Extended Data Fig. 4c**). B2M^EC-cKI^ mice showed enhanced populations of virus-specific tetramer^+^ CD8^+^ T cells, with tetramer^+^ IFNγ and Granzyme B subsets in lungs (Fig. 2o, **Extended Data Fig. 4d**). Correspondingly, B2M^EC-cKI^ mice reduced viral RNA in lungs and improved lung pathology, compared to controls (Fig. 2p, **Extended Data Fig. 4e**). Together, these results demonstrate that MHC-I expression in PMVECs is essential for activating pulmonary resident virus-specific CD8^+^ T cells during later phase of infection, promoting viral clearance and resolution of lung injury.

### PMVECs present H1N1 antigens and stimulate CD8^+^ T cell proliferation *in vitro*

**Figure 3.**
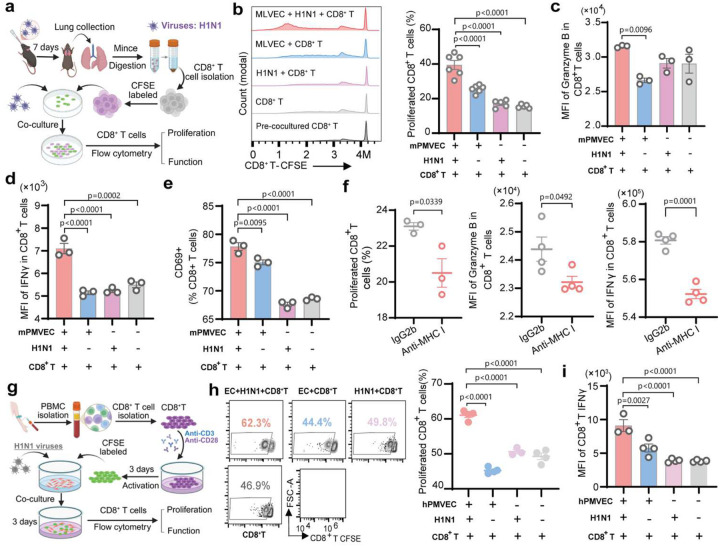
Primary PMVECs directly present viral antigen to CD8+ T cells and induce activation in vitro. Primary mouse (**a-f**) or human (**g-i**) PMVECs were infected with H1N1 (MOI=0.001) and co-cultured with CFSE-prelabeled mouse or human CD8^+^ T cells respectively. Mouse CD8^+^ T cells were isolated from PBS-perfused lungs of C57BL/6 mice at 7 dpi. Human CD8^+^ T cells were sorted from fresh leukopacks. After 3 days of co-culture, CD8^+^ T cell proliferation and effector function were assessed by flow cytometry. (**a**) Scheme of mouse PMVEC-CD8^+^T cell co-culture design. (**b**) Representative flow plots and quantification of mouse CD8^+^ T cell proliferation after co-culture with H1N1-infected or un-infected mouse PMVECs. CD8+ T cells alone or exposed to H1N1 as controls. Effector function of mouse CD8^+^ T cells, including intracellular Granzyme B (**c**), IFNγ (**d**), and surface CD69 (**e**). (**f**) mouse PMVECs were pretreated with anti–MHC-I antibody to block MHC-I antigen presentation. IgG2b as control. CD8^+^ T cell proliferation and effector function (Granzyme B and IFNγ) were measured by flow cytometry. (**g**) Scheme of human PMVEC-CD8^+^ T cell co-culture design. **(h**) Representative flow plots and quantification of human CD8^+^ T cell proliferation. (**i**) Intracellular IFNγ MFI of human CD8^+^ T cells among different groups. Data are presented as mean ± s.e.m. Statistical analysis by unpaired two-tailed t-tests (f) and one-way ANOVA (b-e, h-i).

To test whether PMVECs directly present viral antigens to CD8^+^ T cells, we performed *in-vitro* co-culture assays. Lung CD8^+^ T cells sorted from H1N1-infected C57BL/6 mice (7 dpi) were CFSE (carboxyfluorescein succinimidyl ester)-prelabeled and cultured with primary mouse PMVECs with or without H1N1 infection (Fig. 3a). Viral RNA increased over time in PMVECs (**Extended Data Fig. 5a**), confirming active replication. H1N1 infection upregulated MHC-I expression, as confirmed by RT-qPCR and Western blot analyses (**Extended Data Fig. 5b-c**). Co-culture with infected PMVECs induced strong CD8^+^ T cell proliferation, activation (CD69), and effector function (IFNγ, Granzyme B), compared to all controls (Fig. 3b–e). MHC-I dependence was validated by B2m siRNA knockdown (~40% reduction in MHC- I) and MHC-I antibody blockade, showing reduced CD8^+^ T cell proliferation and effector function (Fig. 3f, **Extended Data Fig. 5d-e**). These findings demonstrate that PMVECs directly present viral antigens via MHC-I to drive CD8^+^ T cell expansion and activation during H1N1 infection. Similar results were observed in human PMVEC coculture assays (Fig. 3g–i). H1N1-GFP (PR8 H1N1) infection increased GFP^+^ primary human PMVECs and MHC-I (HLA-A/B2M) expression at 24 hours post infection (hpi) (**Extended Data Fig. 5f-g**), indicating successful cell infection and activation. Co-culture with infected human PMVECs for 3 days induced more CD8^+^ T cell proliferation and enhanced its IFNγ production (Fig. 3h–i, **Extended Data Fig. 5h**). The increased CD8^+^ T cell proliferation was also observed after coculturing peripheral blood mononuclear cells (PBMCs) with H1N1-infected PMVECs (**Extended Data Fig. 5i**). These findings reveal a conserved PMVEC antigen-presentation mechanism driving CD8^+^ T cell activation in both mice and humans.

### IFNγ enhances endothelial MHC-I expression and CD8^+^ T cell activation

**Figure 4.**
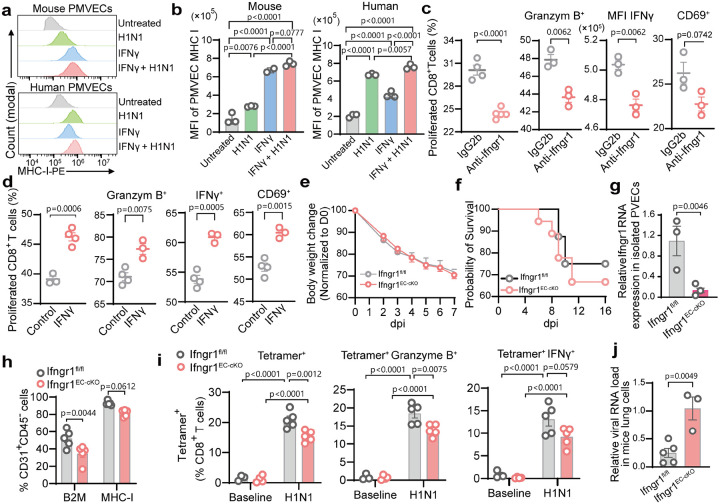
IFNγ enhances EC-mediated CD8+ T cell activation. (**a-d**) In vitro co-culture experiments. (**a**) Representative flow cytometry plots and (**b**) quantification (MFI) of MHC-I expression on mouse or human PMVECs treated with IFNγ, with or without H1N1 infection (MOI=0.001). (**c**) H1N1-infected mouse PMVECs were pretreated with anti-Ifngr1 neutralizing antibody or isotype IgG2b (control) and co-cultured with CD8^+^ T cells. (**d**) mouse PMVECs with or without (control) IFNγ pretreatment were cocultured with CD8^+^ T cell for 3 days. CD8+ T cell proliferation, and effector function (Granzyme B^+^, IFNγ^+^, and CD69+) were measured by flow cytometry. (**e-j**). In vivo of lung endothelial IFNγ signaling. Conditional endothelial-specific IFNγR1 knockout (Ifngr1^fl/fl^:Cdh5-Cre^ERT2^) mice were inoculated with 100 PFU H1N1. Cre^+^ mice (Ifngr1^EC-cKO^) and Cre^−^ controls (Ifngr1^fl/fl^). Lungs were harvested at baseline and 7 dpi. Body weight change (normalized to day 0) (**e**) and survival curve (**f**) of mice following H1N1 infection. (**g**) RT-qPCR quantification of Ifngr1 RNA expression in isolated mouse PVECs at 7 dpi, normalized to GAPDH. (**h**) Flow cytometry analysis of B2M^+^ and MHC-I^+^ PVECs at 7 dpi. (**i**) Flow cytometry analysis of lung resident virus-specific tetramer^+^ CD8^+^, including tetramer^+^ GranzymeB^+^ and tetramer^+^ IFNγ^+^ subsets. (**j**) RT-qPCR of H1N1 NP RNA in lungs, normalized to GAPDH. Data are presented as mean ± s.e.m. Statistical analysis by unpaired two-tailed t-tests (c-d, g-h, j), one-way ANOVA (b), and two-way ANOVA (i)..

IFNγ is a critical regulator of MHC-I on APCs^32^. To assess its role in PMVEC antigen presentation, we treated mouse or human cultured PMVECs with IFNγ, which significantly increased MHC-I protein and mRNA expression (Fig. 4a–b, **Extended Data Fig. 6a**). H1N1 infection combined with IFNγ further enhanced MHC-I, indicating synergy (Fig. 4a–b). IFNγ treatment increased ICAM1 expression in both mouse and human PMVECs, indicating endothelial activation (**Extended Data Fig. 6b-c**). In co-culture assays, H1N1-infected PMVECs strongly upregulated MHC-I expression when exposed to CD8^+^ T cells, and IFNγ pretreatment further boosted MHC-I expression (**Extended Data Fig. 6d-e**). These results suggest a positive feedback loop where CD8^+^ T cell–derived IFNγ enhances PMVEC antigen presentation, driving further CD8^+^ T cell activation. Blocking IFNγ signaling with anti-Ifngr1 reduced CD8^+^ T cell proliferation, CD69 expression, and effector molecule production compared with IgG controls (Fig. 4c). Conversely, IFNγ pretreatment enhanced CD8^+^ T cell proliferation, Granzyme B, IFNγ, and CD69 expression (Fig. 4d), and the same effect also observed in human PMVEC co-cultures (**Extended Data Fig. 6f**). Thus, MHC-1 promotes EC antigen-presenting function in an IFNγ dependent manner.

To assess vivo relevance, we generated inducible EC-specific Ifngr1 knockout mice (Ifngr1^fl/fl^:Cdh5-Cre^ERT2^) (**Extended Data Fig. 6g**). Following H1N1 infection, Ifngr1^EC-cKO^ mice showed higher mortality than controls without significantly difference on body weight loss (Fig. 4e–f). Deletion of Ifngr1 in PVECs was confirmed by RT-qPCR (Fig. 4g). PVECs from Ifngr1^EC-cKO^ mice displayed lower MHC-I, B2M, ICAM1, and VCAM1 expressions, consistent with in vitro results (Fig. 4h, **Extended Data Fig. 6h**). In Ifngr1^EC-cKO^ mice lungs, total CD8^+^ T cells and Granzyme B^+^ CD8^+^ T cells were unchanged (**Extended Data Fig. 6i**), but virus-specific tetramer^+^ CD8^+^ T cells were reduced with impaired IFNγ and Granzyme B expression (Fig. 4i), accompanied by higher viral loads and worsened lung injury, compared to Ifngr1^fl/fl^ mice (Fig. 4j, **Extended Data Fig. 7j**). These results demonstrate that IFNγ signaling in PMVECs is critical for MHC-I–mediated antigen presentation, activation of virus-specific CD8^+^ T cells, and effective antiviral immunity in lungs.

### CD40 enhances PMVEC MHC-I–mediated CD8^+^ T cell activation

**Figure 5.**
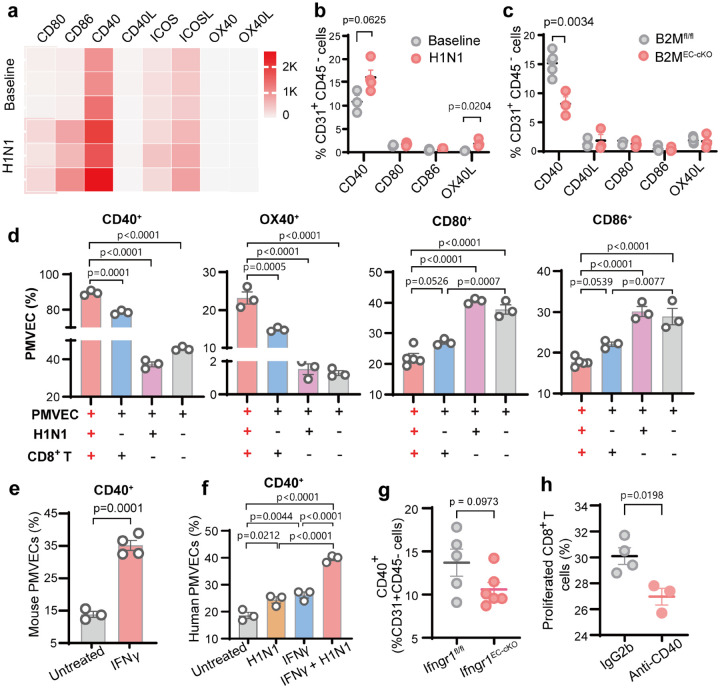
CD40 is a co-stimulatory molecule for MHC-I to facilitate CD8^+^ T cell activation. (**a**) Bulk RNA-seq analysis of sorted tdTomato^+^ PVECs from tdTomato^fl/+^:B2M^fl/fl^:Cdh5-Cre^ERT2^ mice and tdTomato^fl/+^:Cdh5-Cre^ERT2^ mice at baseline and 7 dpi, showing transcripts of CD40, CD80, CD86, CD40L, OX40, OX40L, ICOS, and ICOSL. (**b**) Flow cytometry analysis of surface co-stimulatory molecules in PVECs from C57BL/6 mice at baseline and 7 dpi. (**c**) Comparison of co-stimulatory molecules in PVECs from ^B2MEC-cKO^ and B2M^fl/fl^ mice at 7 dpi by flow cytometry. (**e-f, h**) In vitro co-culture assays. (**d**) Flow cytometry analysis of CD40, CD80, CD86, and OX40L expression on PMVECs after co-culture with pulmonary CD8^+^ T cells from C57BL/6 mice at 7 dpi. Flow cytometry analysis of CD40 expression on mouse (**e**) and (**f**) human PMVECs, treated with or without IFNγ, and/or H1N1 infection. (**g**) Flow cytometry analysis of CD40 expression on PVECs from Ifngr1^EC-cKO^ and Ifngr1^fl/fl^ mice at 7 dpi. (**h**) CD40 blockade: H1N1-infected PMVECs were pre-treated with anti-CD40 or IgG2b control were co-cultured with CD8^+^ T cells; CD40 levels measured by flow cytometry. Data are presented as mean ± s.e.m. Statistical analysis by unpaired two-tailed t-tests (b-c, e, g-h), one-way ANOVA (d, f).

To assess co-stimulatory molecules in PMVEC–mediated antigen presentation, we analyzed bulk RNA-seq data of tdTomato^+^CD31^+^/CD45− PVECs sorted from tamoxifen-injected tdTomato^fl/+^:B2m^fl/fl^:Cdh5-CreERT2 mice at baseline and 7 dpi. Bulk RNA-seq showed that CD40, CD80, and CD86 were significantly upregulated after H1N1 infection (Fig. 5a). However, the flow cytometry confirmed induction of CD40^+^ and OX40L^+^ PVECs at 7 dpi with no change on CD80^+^ and CD86^+^ PVECs (Fig. 5b) in C57BL/6 mice. In B2M^EC-cKO^ mice, only CD40^+^ PMVECs were reduced, linking MHC-I integrity to CD40 expression (Fig. 5c). *In vitro*, H1N1 infection alone did not increase CD40^+^ and OX40L^+^ PMVECs, but co-culture with CD8^+^ T cells robustly upregulated both, indicating dependence on T cell-derived signals (Fig. 5d). IFNγ treatment robustly increased CD40^+^ cells in both mouse and human PMVECs (Fig. 5e–f), while endothelial Ifngr1 deletion reduced CD40^+^ PVECs (Fig. 5g). Thus, CD40 induction in PMVECs depends on IFNγ rather than direct viral infection. Functionally, CD40 blockade in H1N1-infected PMVEC–CD8^+^ T cell co-cultures significantly reduced CD8^+^ T cell proliferation (Fig. 5h), indicating that CD40 provides essential co-stimulation during PMVEC antigen presentation. Furthermore, we compared the immune profiles of PMVECs and Dendritic cells (DCs, professional APCs) using transcriptomic analysis of scRNA-seq of C57BL/6 lung cells at baseline and 4, 7, and 10 dpi (**Extended Data Fig. 2a &7a-g**). We identified “immune PMVECs” based on differential expression of MHC molecules & regulators and cytokines (**Extended Data Fig. 7a-g**), using the same strategy as before^22^. Comparing to DCs, immune PMVECs showed higher MHC-I expression but produced fewer cytokines (**Extended Data Fig. 7h-i**). Notably, H1N1 induced relative lower co-stimulatory molecules but higher co-inhibitory molecules such as CD279 (**Extended Data Fig. 7j-k**), suggesting PMVEC antigen presentation is different from DC professional antigen presentation.

### IFNγ–STAT1 signaling mediates PMVEC MHC-I upregulation

**Figure 6.**
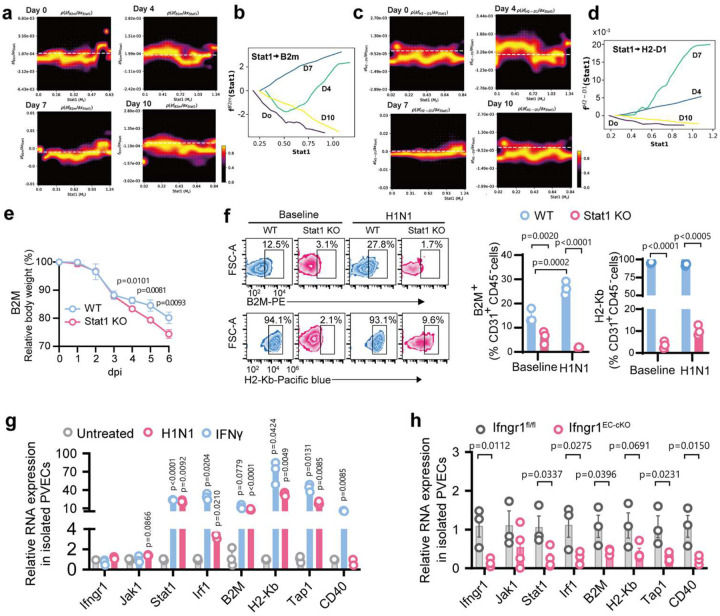
IFNγ-STAT1 signaling drives MHC-I upregulation in PMVECs during H1N1 infection. (**a-d**) scRNA-seq analysis of PVECs from C57BL/6 mice at baseline, 4, 7, and 10 dpi. Instaneous RNA velocity and transcriptional vector field of mixed single PVECs from different time points was reconstructed using Dynamo and GraphVelo to reveal cell-state transition between different time points. The gene regulatory information was obtained from the velocity vector by Jacobian regulation analysis to reveal how changing the expression of one gene, the regulator, affects the velocity of an effector gene (dose response curve). The x-axis is the expression of the regulator - Stat1 and the y-axis is the jacobian of the effector genes – B2m (**a**) and H2-D1 (**c**) with respect to the regulator Stat1. Positive values indicate activation while negative values indicate inhibition. The cumulative Stat1 regulation over time for B2m (**b**) and H2-D1 (**d**), also called dose response. (**e-f**) Global *Stat1* knockout (KO) mice and wild-type (WT) mice were inoculated with 100 PFU H1N1. (**e**) Body weight change. (**f**) Representative flow plots of B2M and H2-Kb and quantification of B2M and H2-Kb surface expression on PVECs; uninfected mice as baseline. (**g**) RT-qPCR of type II IFN pathway gene in cultured mouse PMVECs treated with IFNγ or H1N1, compared to untreated/uninfected controls. (**h**) Ifngr1^EC-cKO^ and Ifngr1^fl/fl^ mice were infected with H1N1; PVECs were isolated at 7 dpi. RT-qPCR of *Ifngr1*, Jak1, *Stat1*, and Stat1’s downstream targets (*Irf1, H2-Kb, Tap1, and CD40*). Data are presented as mean ± s.e.m. Statistical analysis by unpaired two-tailed t-tests (g-h) and two-way ANOVA (f).

Single-cell transcriptomics analysis revealed dynamic transcriptional changes and cell-state transitions in PMVECs of C57BL/6 lung cells at baseline, 4, 7, and 10 dpi. The RNA velocity vector field analysis using GraphVelo 33 and Dynamo 34 showed that PMVECs from uninfected mice remained transcriptionally steady, whereas PMVECs from infected mice displayed progressive activation (**Extended Data Fig. 8a**). Within the vector field, we perform Jacobian regulation analysis^34^ to reveal how changing the expression of one gene, the regulator, affects the mRNA turnover dynamics of an effector gene (i.e., a dose response curve). Jacobian analysis identified Stat1 as a key regulator of MHC 1 genes, including B2M and H2-D1 (Fig. 6a–d), as well as Ifngr1, H2-K1, H2-T23, and H2-D4 (**Extended Data Fig. 8b**), with the regulatory effect peaking at 7 dpi. These findings suggest that PMVECs upregulate antigen presentation machinery in a STAT1-dependent manner following H1N1 infection.

To confirm the critical role of IFNγ–STAT1 signaling in regulating MHC-I expression, global Stat1 knockout mice infected with H1N1 showed greater weight loss and diminished MHC-I (B2M, H2-Kb) expression in PMVECs compared to controls (Fig. 6e–f). *In vitro*, Stat1 knockdown in cultured mouse PMVECs decreased antigen-presentation molecules (B2M, H2-Kb, Tap1, CD40) and IFN II pathway genes (Stat1, and Irf1) (**Extended Data Fig. 8c**); IFNγ treatment or H1N1 infection strongly upregulated these genes (Fig. 6g) and endothelial-specific Ifngr1 knockout (Ifngr1^EC-cKO^) mice showed reduced Stat1 activation and lower MHC-I and antigen-processing genes following infection (Fig. 6h). These results demonstrate that IFNγ–STAT1 regulates PMVEC-CD8^+^ T cells positive feedback loop to escalate PMVEC antigen-presenting and CD8^+^ T cell activation during viral infection.

### Impaired PMVEC antigen presentation contributes to H5N1 pathogenicity

**Figure 7.**
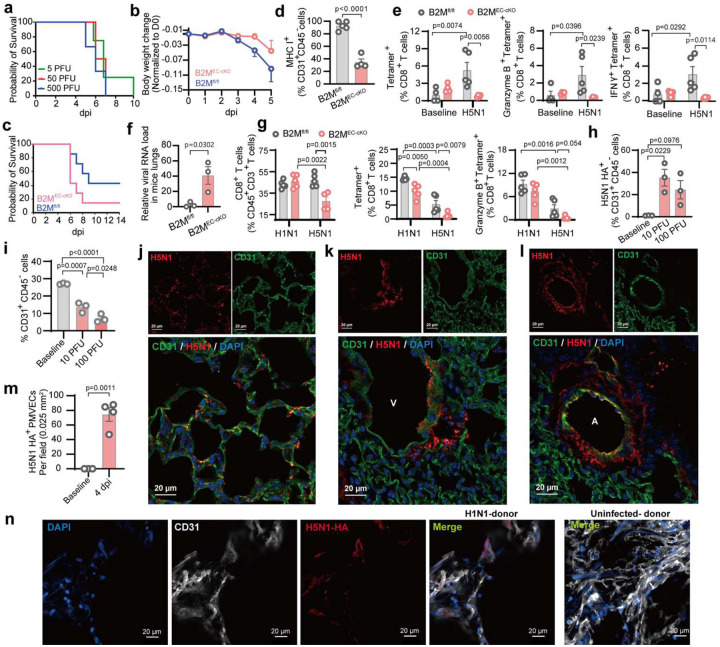
Endothelial MHC-I promotes CD8^+^ T cell activation for viral clearance during H5N1 infection. (**a**) Survival of C57BL/6 mice inoculated intranasally with an emerging human H5N1 strain (A/Texas/37/2024) at 5, 50, or 500 PFU. (**b-f**) The B2M^EC-cKO^ and B2M^fl/fl^ mice were inoculated with 5 PFU H5N1. (**b**) Body weight change and (**c**) survival curve of mice. (**d**) MHC I^+^ PVECs by flow cytometry. (**e**) Lung-resident virus-specific CD8+ T cells: tetramer^+^, Granzyme B^+^ tetramer^+^, IFNγ^+^ tetramer^+^ subsets by flow cytometry. (**f**) Viral RNA quantification in lungs by RT-qPCR, normalized to GAPDH. (**g**) Comparative analysis of pulmonary CD8^+^ T cell responses in B2M^EC-cKO^ and B2M^fl/fl^ mice infected with 10 PFU H5N1 vs 10 PFU H1N1, including total resident CD8^+^ T, virus-specific tetramer^+^ CD8^+^ T cells, and Granzyme B^+^ Tetramer^+^ CD8^+^ T cells. (**h-m**) C57BL/6 mice were inoculated with H5N1. Flow cytometry of (**h**) H5N1 HA^+^ PVECs and (**i**) total PVECs from C57BL/6 mice following infection with 10 or 100 PFU at 4 dpi. (**j-l**) Representative confocal images of lung sections stained with CD31 (ECs) and H5N1-HA at 4 dpi for microvasculature (**j**), Venule (**k**), and Arteriole (**l**). A: Arteriole, V: Venule, B: Bronchus. (**m**) Quantification of the mean percentage of H5N1^+^CD31^+^ ECs (PMVEC, Venule and Arteriole ECs) among total CD31^+^ cells within infected regions, based on three representative images. (**n**) Representative confocal images of H5N1-infected PMVECs in human PCLSs. Data are presented as mean ± s.e.m. Statistical analysis by unpaired two-tailed t-tests (d, f, m), one-way ANOVA (h-i), and two-way ANOVA (e, g).

The emergent HPIAV H5N1 strain (human A/Texas/37/2024)^35^ was used to assess the role of PMVEC antigen presentation in antiviral defense against highly pathogenic influenza viruses. Survival analysis in C57BL/6 mice showed dose-dependent lethality and high pathogenicity of this strain (Fig. 7a, **Extended Data Fig. 9a**). To dissect the contribution of PVEC MHC-1 antigen presentation, B2M^EC-cKO^ and B2M^fl/fl^ mice were infected with 5 PFU H5N1. B2M^EC-cKO^ mice exhibited greater weight loss and increased mortality, compared with controls (Fig. 7b–c). Flow cytometry confirmed loss of MHC-I on PVECs in B2M^EC-cKO^ mice at 6 dpi (Fig. 7d), correlating with reduced lung-resident CD8^+^ T cells, including Granzyme B^+^ and IFNγ^+^ subsets (**Extended Data Fig. 9b**), as well as virus-specific tetramer^+^ CD8^+^ T cells and their effector populations (tetramer^+^ IFNγ^+^, and tetramer^+^ Granzyme B^+^ CD8^+^ T cells (Fig. 7e). Correspondingly, viral RNA levels were elevated in B2M^EC-cKO^ lungs at 6 dpi (Fig. 7f), and histological analysis revealed worsened lung inflammation (**Extended Data Fig. 9c**), indicating that PVEC MHC-I is essential for CD8^+^ T cell–mediated viral clearance and lung protection during H5N1 infection. Direct comparison of H5N1 and H1N1 infections (10 PFU) at 4 dpi showed that endothelial MHC-I deficiency caused a more pronounced reduction in both total and effector CD8^+^ T cells in B2M^EC-cKO^ mice lungs during H5N1 infection (Fig. 7g, **Extended Data Fig. 9d**), highlighting the important role of PVEC antigen presentation in controlling H5N1 infection. Interestingly, H5N1-infected B2M^fl/fl^ mice exhibited higher effector CD8^+^ T cells in lungs but weaker virus-specific responses compared with H1N1-infected controls (Fig. 7g, **Extended Data Fig. 9d**), suggesting that despite broader recruitment, functional antiviral responses are limited.

To evaluate PMVEC susceptibility, H5N1 infections at 10 or 100 PFU were performed in C57 mice and compared with H1N1. Flow cytometry showed ~35% H5N1^+^ PVECs at 10 PFU, significantly higher than H1N1^+^ PVECs at 100 PFU at 4 dpi (Fig. 7h, Fig. 1g). At 100 PFU, infected EC counts slightly decreased, likely due to death of heavily infected cells, as reflected by lower total PMVEC counts (Fig. 7i). Confocal imaging further showed high population of H5N1-infected PMVECs in infected areas at 4 dpi, compared with H1N1 (Fig. 7j, 7m, **Extended Data Fig. 9e**). Notably, H5N1 infected not only PMVECs but also arteriole and venous ECs (Fig. 7k–l; **Extended Data Fig. 9f-g**), whereas H1N1 infected only PMVECs (Fig. 1d–e, **Extended Data Fig. 1h-k**). These findings suggest that impaired activation of viral-specific CD8^+^ T cells, early infection of macrovascular ECs, and a high population of infected PMVECs contribute to H5N1’s systemic dissemination and high pathogenicity. Similarly, human PCLSs infected with H5N1 (1000 PFU) showed virus-infected PMVECs, consistent with murine lung tropism (Fig. 7n).

## DISCUSSION

Our study establishes pulmonary microvascular endothelial cells (PMVECs) as critical modulators of antiviral immunity during IAV infection. We show that H1N1 directly infects pulmonary vascular ECs (PVECs), supporting evidence that ECs are susceptible to viruses, e.g., SARS-CoV-2^36–38^. Notably, H1N1 infection of ECs is restricted to PMVECs and does not affect lung arteriolar and venous ECs. Infected PMVECs activate resident CD8^+^ T cells by cross-presenting viral antigen, highlighting their immunomodulatory role in antiviral response and lung injury. These observation across *in vitro*, *ex vivo*, and *in vivo* models reinforces our previous identification of the functional sub-populations– immune ECs^22,39^. Using EC-specific B2M knock-down and knock-in mice, we confirmed that MHC-I on PMVECs is essential for antiviral CD8^+^ T cell responses, with CD40 as a key co-stimulatory molecule. Our study also suggests that viral specific IFNγ^+^ CD8^+^ T cells and Granzyme B^+^ CD8^+^ T cells activated by PMVECs are required for viral clearance and lung inflammation resolution.

The temporal dynamics of lung endothelial infection further highlight their role in sustaining adaptive immunity. H1N1 infects PMVECs during later stages of lung injury, starting from day 4 and reaching maximal infection at 7 dpi, whereas epithelial cells and DCs are infected earlier^40^. This temporal distinction suggests that the initial CD8^+^ T cell activation and antigen presentation may be driven by DCs, whereas sustained and prolonged T cell responses to clear viruses rely on PMVEC-mediated antigen presentation. scRNA-seq analysis of immune PMVECs and DCs revealed that, following H1N1 infection, PMVECs express higher MHC-I but lower inflammatory cytokines than DCs. Although classical co-stimulatory molecules such as CD80 and CD86^13,18,41,42^ are expressed at low levels, CD40 is upregulated on PMVECs after infection, as confirmed *in vivo* and *in vitro*. This suggests a distinct co-stimulatory mechanism in endothelial antigen presentation that sustains adaptive immunity for virus clearance.

Mechanistically, we identify IFNγ as a central regulator of endothelial immunity. IFNγ from activated CD8^+^ T cells increase MHC-I and CD40 expression on mouse and human PMVECs via endothelial IFNγR1, promoting antigen presentation and expansion of functional CD8^+^ T cells (IFNγ^+^ and Granzyme B^+^) in a positive feedback loop (**Extended Data Fig. 10**). RNA velocity reconstruction showed that PMVECs transition from quiescent to activated transcriptional state upon infection, with Stat1 driving antigen-presenting gene induction. Stat1 knockdown in cultured PMVECs or global Stat1 knockout *in vivo* reduced MHC-I and CD40 in PVECs, whereas IFNγ induced Stat1 and its targets; endothelial Ifngr1 deletion abolished these effects. These data establish the IFNγ–Stat1 axis as a critical regulator of PMVEC antigen presentation and antiviral CD8^+^ T cell responses, which is consistent with prior studies in other cell types^43,44^.

The emerging HPAIV H5N1 “bird flu” poses a potential pandemic threat to public health, due to its high mortality and cross-species transmissibility^45–48^. One fundamental question that has remained unanswered is why this influenza virus is far more pathogenic than the more conventional influenza A virus. We observed that PMVEC antigen presentation shapes the outcome of highly pathogenic influenza virus (HPAIV) infection. Using the emergent H5N1 strain (human A/Texas/37/2024), we show that loss of endothelial MHC-I reduces total and effector CD8^+^ T cells, including Granzyme B^+^ and IFNγ^+^ subsets, and increases viral loads and lung injury, suggesting the essential role in viral defense of endothelial MHC-I. Although H5N1 recruited more CD8^+^ T cells than H1N1, virus-specific effector responses were limited, indicating broad but functionally weak T cell immunity. Furthermore, H5N1 infects a large population of PMVECs early in infection and additionally targets a large proportion of vascular ECs, likely contributing to early systemic dissemination^7^. In contrast, H1N1 infection is confined to PMVECs during late-stage lung injury. Thus, although PMVECs serve as a conserved platform for antigen presentation, their ability to activate virus-specific CD8^+^ T cells is impaired in H5N1 infection, likely contributing to H5N1’s high pathogenicity as well. Overall, our findings imply PMVEC immunomodulation as a determinant for H5N1 pathogenicity and highlight immunomodulatory PMVECs as therapeutic targets.

Several studies have suggested that endothelial cells can act as non-professional antigen-presenting cells, but this raises the fundamental question of whether such “non-professional” antigen presentation is relevant for pathogenesis and host defense. We demonstrate that lung microvascular endothelial antigen presentation is indeed a core component of the host response to influenza infections. Our findings identify PMVECs as previously unrecognized essential mediators of antiviral immunity, as they directly present viral antigens and activate CD8^+^ T cells, highlighting the EC–T cell axis as a critical modulator of host defense and a potential therapeutic target in severe influenza and other viral pneumonia.

Our study has several limitations. First, due to the H5N1 reporter virus we generated (H5N1-Cre) failed to label H5N1-infected PVECs in vivo, we used H5N1 antibodies to quantify H5N1-infected PVECs through flow cytometry and confocal imaging. However, considering the specificity and sensitivity of these antibodies, the quantification of H5N1-infected PVECs might be either over-counted or under-counted. Secondly, while our study focused on influenza, it remains unclear whether lung EC antigen presentation represents a generalizable antiviral mechanism. Testing other respiratory viruses, such as SARS-CoV-2, will be important. Thirdly, although our findings suggest that ECs contribute to CD8^+^ T cell responses at later stages of infection, their role in comparison to professional antigen-presenting cells – dendritic cells in initiating versus amplifying immunity requires further investigation. This could potentially be assessed through time-resolved tracking of T cell activation. Finally, we did not investigate the potential pathological consequences of prolonged EC–T cell interactions; future studies should examine whether sustained activation contributes to lung injury. These limitations outline key directions for future research to clarify both protective and pathological roles of EC antigen presentation during respiratory viral infections.

## MATERIALS AND METHODS

### Mice and ethics statement.

C57BL/6, Rosa26-LSL-tdTomato, B2Mfl/fl, Rosa26B2M-stopfl/fl, Ifngr1fl/fl, Cdh5-CreERT2, Stat1 global knockout mice were obtained from Jackson Laboratory. Endothelial cell–specific knockout and knock-in mouse models were generated by crossing Cdh5-CreERT2 mice with B2Mfl/fl, Rosa26LSL-B2M, or Ifngr1fl/fl mice. To induce gene deletion or overexpression specifically in ECs, tamoxifen was administered via intraperitoneal injection for 2 consecutive days. Experiments were performed approximately 30 days after tamoxifen. All mice were on a C57BL/6 background and housed under specific pathogen–free conditions. Genotyping was done by PCR on DNA from tail samples to verify Cre, floxed, knockout, or knock-in alleles. Only mice with confirmed genotypes were used in experiments. Gene modification efficiency was confirmed by qPCR, flow cytometry, and Western blot of CD31^+^ pulmonary Vascular ECs (PVECs). All animal experiments were approved by the Institutional Animal Care and Use Committee (IACUC) of University of Pittsburgh and conducted in accordance with NIH guidelines.

### Biosafety information.

All work with live H5N1 was approved by the institutional biosafety committee (IBC) and performed at BSL-3 in the University of Pittsburgh Regional Biocontainment Laboratory and at Texas Biomedical Research Institute, following strict safety protocols including powered air-purifying respirators, class III biosafety cabinets, and individually ventilated caging. All wastes and animal materials were appropriately disinfected, sterilized, or inactivated, and the facility requires full clothing changes and showers for entry and exit. The RBL is registered with the CDC and USDA for H5N1 research, and all inactivation procedures were approved by the university biosafety committee.

### Cell cultures.

All cell lines and primary cells used in this study were purchased from the American Type Culture Collection (ATCC) and Lonza. Madin-Darby Canine Kidney (MDCK) cells, used for influenza virus propagation and titration, were cultured in Dulbecco’s Modified Eagle Medium (DMEM, Gibco, Cat# 11965092) supplemented with 10% FBS (Gibco, Cat# 16000044), 2 mM L-glutamine (Gibco, Cat# 25030081) and 1% antibiotics (penicillin 100 U/mL and streptomycin 100 μg/mL, Gibco, Cat# 15140122). Murine pulmonary microvascular endothelial cells (PMVECs) were cultured in EGM-2 MV Mouse Endothelial Cell Growth Medium Kit (Lonza, Cat# CC-3156) supplemented with the SingleQuots^™^ Kit of growth factors and supplements (Lonza, Cat# CC-4147). Human lung microvascular endothelial cells (hLMVECs) were cultured in EGM-2 MV Human Endothelial Cell Growth Medium Kit (Lonza, Cat# CC-3121) with the corresponding SingleQuots^™^ Kit (Lonza, Cat# CC-4133). Isolated human and mouse CD8^+^ T cells were maintained in RPMI 1640 medium (Gibco, Cat# 11875093) supplemented with 10% FBS, 1% penicillin-streptomycin, and 2 mM L-glutamine. All these cells were maintained at 37°C in a humidified incubator with 5% CO4_2_.

### IAV modification and propagations.

Cre-expressing recombinant IAV including H1N1-Cre viruses were constructed by inserting the Cre recombinase gene into the NS segment of the A/PR/8/34 (H1N1), using reverse genetics systems^49^. The H1N1 (A/PR/8/34), H5N1 (A/Texas/37/2024), and H1N1-Cre were propagated in MDCK cells. Upon reaching 90% confluence, cells in T75 flasks were washed with phosphate-buffered saline (PBS) and infected with IAVs (MOI= 0.001) in serum-free DMEM (Sigma-Aldrich, Cat# T1426). After 1 hour of viral adsorption at 37°C, the inoculum was removed and replaced with fresh infection medium containing 2 μg/mL TPCK-trypsin (Thermal Fisher, Cat# 20233). At 48 hpi, supernatants containing virus were harvested, clarified by centrifugation at 2,000 × g for 10 minutes, aliquoted, and stored at −80°C. Viral titers were determined by plaque assay in MDCK cells.

### Virus titration using immunostaining.

MDCK cells were cultured at the density of 10^6^ cells per well in 6-well plates and kept overnight at 37°C in a humidified 5% CO_2_ incubator. The next day, confluent MDCK monolayers were infected with serial viral dilutions for 1 h at 37°C. Following viral adsorption, cell monolayers were overlaid with post-infection media containing agar and incubated at 37°C in a humidified 5% CO_2_ incubator. At 24 hours post-infection (hpi), cells were fixed overnight with 10% neutral buffered formalin solution. Cells were then permeabilized with 0.5% (v/v) Triton X-100 in PBS for 15 min at RT and immunostained using an influenza virus anti-NP mouse monoclonal antibody (MAb) HT103 (1:100) and the Vectastain ABC kit (Vector Laboratories), following the manufacturers’ instruction.

### Pulmonary ECs and mouse model of IAV Infection.

Primary mouse and human PMVECs were seeded in 6-well plates until 90% confluence. Cells were infected with H1N1 at a MOI of 0.001 for 1 hour at 37°C, then washed with PBS and cultured in fresh medium for the indicated time. Viral RNA was measured by qPCR targeting the NP gene, and viral protein expression was evaluated by flow cytometry using an anti-NP antibody (Bio-techne, Cat# MAB113961). Age- and sex-matched C57BL/6, Rosa26-LSL-tdTomato, EC-B2M KO, EC-B2M KI, EC-Ifngr11 KO, and control mice were lightly anesthetized with isoflurane and intranasally inoculated with 100 PFU H1N1 or 5 PFU H5N1 in 40 μL PBS. Body weight and survival were monitored daily. At 7 dpi, lung tissues were harvested for virological, immunological, and histological analysis.

### Ex-vivo model of human precision-cut lung slices (PCLS) for IAV Infection.

PCLS were prepared from fresh human lung tissue to create a physiologically relevant *ex vivo* model that preserves the lung’s native structure and cellular diversity^50^. Briefly, lung lobes were cannulated and gently inflated through the airways with 1.5% low-melting-point agarose (Sigma-Aldrich, Cat# A9414) in PBS. After the agarose solidified on ice, the tissue was sliced into 200–300 μm sections using a vibratome (Leica VT1200S). The PCLS were then transferred into 24-well plates, which were maintained in 1 mL DMEM/F-12 medium (GIBCO, Cat# 11320033) supplemented with 100 U/ml of penicillin, and 100 μg/ml streptomycin. The lung slices in each well were infected with 1 × 10^5^ PFU H1N1 IAV (A/PR/8/34) for 1 hour. Virus diluent was used as a negative control. After incubation, the virus was removed and washed with PBS, and fresh medium was added. Supernatants were collected at indicated time for viral RNA analysis, and slices were harvested at 48 hpi for flow cytometry and immunofluorescence.

### Single cell capture and library preparation.

The C57BL/6 mice were inoculated with H1N1 PR8 100 PFU. The lungs were harvested, digested and dissociated at baseline, and 4, 7 and 10 dpi. The lung cells were mixed from 3 mice at each time point. The DAPI- lung cells were sorted by FACS and loaded into a 10x Genomics microfluidics chip and encapsulated with barcoded oligo-dT-containing gel beads using the 10x Genomics Chromium controller according to the manufacturer’s instructions. Cell viability at each time point was > 80% with a target of sequencing 5,000 cells at each time point were targeted. Single-cell libraries were constructed using v2 kit according to the manufacturer’s instructions. cDNA quality of each time points was checked (high quality). Libraries from individual samples were multiplexed into two lanes for sequencing on NovaSeq 6000 S4 (~100k reads per cell and per lane 2×150bp Paired-Read) at a depth of ~200,000 reads per cell.

### Processing the scRNA-seq data and identifying cell types from scRNA-seq data.

For each timepoint, Cellranger count (v8.0.1) was run on the raw fastq files and then velocyto (v0.17.17) was run to obtain unspliced and spliced RNA counts for RNA velocity analysis. To identify specific cell types within the datasets, all the timepoints were aggregated, the raw unspliced and spliced counts were processed with the recipe_monocle function (n_top_genes=2000), PCA was performed to obtain a 30-dimensional PCA space and then UMAP was performed to obtain a 2-dimensional embedding. With the UMAP embedding, leiden clustering was performed using scanpy (v1.11.2) (resolution = 0.25) to obtain clusters. Using cell type specific marker genes for endothelial cells (Pecam1+ and Cdh5+), Cd8+ T-cells (Cd8a+, Cd3e+, Cd3g+), Macrophages (Cd68+, Itgam+, Ccr2+, Arg1+) and Dendritic cells (Cd209a+, cDC1: Itgax+, Cd8a+, Cd68+, Cd80+ and cDC2: Xcr1+, Clec9a+, Ly75+, Itgae+) various cell types were identified and subset for downstream analysis.

### Cell-cell communication analysis.

For each timepoint, the raw spliced count matrices for only cells identified as either endothelial or Cd8+ T-cells were used as input to the CellChat^27^ R package (v2.2.0) along with the corresponding identified cell type labels. The counts were then normalized, and significant cell-cell signals were obtained. The cell-cell communication probability was calculated with the computeCommunProb function (type = truncatedMean, trim = 0.05). The significant cell signaling pathways were then analyzed between the different time points.

### RNA velocity correction and vector field reconstruction with dynamo.

Instantaneous RNA velocity for a gene was calculated using both the unspliced (mRNA contains introns) and spliced (mRNA without introns) mRNA counts. Thus, each cell data point was represented as a vector of gene expression and a vector of RNA velocities. Dynamo^34^ was used to learn a continuous vector field that maps the gene expression space to the RNA velocity space, representing the gene-gene regulation of the system. For genes with low unspliced or spliced expression inappropriate for RNA velocity inference. We obtained their velocities using GraphVelo^33^ (v 0.1.9) which identifies a subset of genes whose velocities are highly confident and with this information refines the velocity vectors of the remaining genes. First, from the larger dataset, containing all the time points, only endothelial cells were selected. The data was then normalized and then UMAP and leiden clustering was performed. Cell clusters that clustered far away from most cells were removed and then the data was separated into individual timepoints (0, 5, 7, 10 days). The dynamics and velocity were then calculated and then GraphVelo was applied to correct velocities. The time points were aggregated for vector field reconstruction.

### Regulation analysis.

With the vector field, gene regulatory information was obtained using dynamo Jacobian analysis^34^. The analysis revealed how changing the expression of one gene, the regulator, affects the transcription rate of an effector gene. A positive value indicates activation, while a negative value indicates inhibition. From the Jacobian, we then reconstructed the effective regulator dose - effector response curve. It was obtained by binning the cells based on the regulator mRNA level, and averaging the Jacobian value within each bin, then performing numerical integration of the average Jacobians over the regulator mRNA level. From the larger dataset, containing all the time points, only endothelial cells were selected. The data was then normalized and then UMAP and leiden clustering was performed. Cell clusters that clustered far away from most cells were removed and then the data was separated into individual timepoints (0, 5, 7, 10 days). The RNA velocities were then calculated followed with velocity correction using GraphVelo. The vector field for each time point was then reconstructed. The Jacobian regulation information was then obtained from the individual vector fields. While reconstructed from scRNA-seq data, the differences of the Stat1 mRNA-effector transcription dose-response curves at different days reflects the nuclear Stat1 level change between the resting state and activation of the Jak-Stat1 pathway through Stat1 phosphorylation. For each time point, raw count matrices were generated with Cell Ranger (v3.0.0). Data were analyzed in Seurat (v2.3) separately for each time point. Normalization and scaling were performed with *NormalizeData* and *ScaleData*. Variable genes were identified using *FindVariableGenes* (x.low.cutoff=0.0125, x.high.cutoff=3, y.cutoff=0.5). Principal component analysis (PCA) was run on these genes using *RunPCA*. Cell clusters were identified with *FindClusters* (top 20 PCs, k.param=10, resolution=0.4) and visualized by uniform manifold approximation (UMAP) (*RunUMAP*). Marker genes for each cluster were identified with *FindMarkers* (only.pos=TRUE), with multiple testing correction by the Benjamini–Hochberg method.

### Statistical Analysis.

Data are shown as mean ± s.e.m from at least three independent experiments. GraphPad Prism 10.0 software was used for analysis. For two-group comparisons, two-tailed Student’s T-test was used. For three or more groups, one-way or two-way ANOVA with multiple comparison tests was performed, and two-way ANONA was corrected by Tukey post-hoc test. A p-value of less than 0.05 was considered significant. Details of tests used are in the figure legends. *:p < 0.05, **:p < 0.01, ***:p < 0.001, and ****:p < 0.0001.

## Supplementary Material

Supplementary Files

This is a list of supplementary files associated with this preprint. Click to download.

- Supplementalinformationfinal.docx
- Extendeddata12.16.2025.pdf

Supplementary Information is available for this paper.

## Data Availability

The scRNA-seq data generated for this study have been made publicly available via NCBI-GEO GSE201541.
